# Changes in peripheral arterial blood pressure after resuscitative endovascular balloon occlusion of the aorta (REBOA) in non-traumatic cardiac arrest patients

**DOI:** 10.1186/s12873-021-00551-y

**Published:** 2021-12-15

**Authors:** Jostein Rødseth Brede, Eivinn Skjærseth, Pål Klepstad, Trond Nordseth, Andreas Jørstad Krüger

**Affiliations:** 1 Department of Emergency Medicine and Pre-Hospital Services, St. Olav’s Hospital, Trondheim University Hospital, Trondheim, Norway; 2 Department of Research and Development, Norwegian Air Ambulance Foundation, Oslo, Norway; 3 Department of Anesthesiology and Intensive Care Medicine, St. Olav’s Hospital, Trondheim University Hospital, Trondheim, Norway; 4 Department of Circulation and Medical Imaging, Faculty of Medicine and Health Sciences, Norwegian University of Science and Technology (NTNU), Trondheim, Norway

**Keywords:** Aortic occlusion, Advanced cardiovascular life support, Resuscitation, REBOA, Blood pressure

## Abstract

**Background:**

Resuscitative endovascular balloon occlusion of the aorta (REBOA) may be an adjunct treatment to cardiopulmonary resuscitation (CPR). Aortic occlusion may increase aortic pressure and increase the coronary perfusion pressure and the cerebral blood flow. Peripheral arterial blood pressure is often measured during or after CPR, however, changes in peripheral blood pressure after aortic occlusion is insufficiently described. This study aimed to assess changes in peripheral arterial blood pressure after REBOA in patients with out of hospital cardiac arrest.

**Methods:**

A prospective observational study performed at the helicopter emergency medical service in Trondheim (Norway). Eligible patients received REBOA as adjunct treatment to advanced cardiac life support. Peripheral invasive arterial blood pressure and end-tidal CO_2_ (EtCO_2_) was measured before and after aortic occlusion. Differences in arterial blood pressures and EtCO_2_ before and after occlusion was analysed with Wilcoxon Signed Rank test.

**Results:**

Five patients were included to the study. The median REBOA procedural time was 11 min and median time from dispatch to aortic occlusion was 50 min. Two patients achieved return of spontaneous circulation. EtCO_2_ increased significantly 60 s after occlusion, by a mean of 1.16 kPa (*p* = 0.043). Before occlusion the arterial pressure in the compression phase were 43.2 (range 12–112) mmHg, the mean pressure 18.6 (range 4–27) mmHg and pressure in the relaxation phase 7.8 (range − 7 – 22) mmHg. After aortic occlusion the corresponding pressures were 114.8 (range 23–241) mmHg, 44.6 (range 15–87) mmHg and 14.8 (range 0–29) mmHg. The arterial pressures were significant different in the compression phase and as mean pressure (*p* = 0.043 and *p* = 0.043, respectively) and not significant in the relaxation phase (*p* = 0.223).

**Conclusion:**

This study is, to our knowledge, the first to assess the peripheral invasive arterial blood pressure response to aortic occlusion during CPR in the pre-hospital setting. REBOA application during CPR is associated with a significantly increase in peripheral artery pressures. This likely indicates improved central aortic blood pressure and warrants studies with simultaneous peripheral and central blood pressure measurement during aortic occlusion.

**Trial registration:**

The study is registered in ClinicalTrials.gov (NCT03534011).

## Background

Resuscitative endovascular balloon occlusion of the aorta (REBOA) may increase the aortic pressure during cardiopulmonary resuscitation (CPR) and can possibly be an adjunct treatment in non-traumatic cardiac arrest patients. The use in preclinical and human studies has recently been reviewed [1, 2] and the procedure is shown feasible during pre-hospital CPR [3]. However, the effects of REBOA on central aortic or peripheral blood pressure (BP) during CPR in humans is scarcely described [4–8]. Case reports indicate that REBOA may increase aortic pressure and subsequently the coronary perfusion pressure (CPP) [4, 7]. Studies on CPR without REBOA suggest that changes in radial arterial pressure may indicate changes in central pressure [9, 10].

The central aortic BP is the main determinant for the CPP during resuscitation. Increase in CPP is associated with return of spontaneous circulation (ROSC) in humans [11]. The REBOA catheter currently in use in Norway is not approved for aortic BP measurements, which prevents the direct measure of central aortic pressure. The CPP is the difference between the relaxation phase pressure in the aorta and the right atrial pressure. Hence, to increase the relaxation phase aortic pressure by applying REBOA will likely increase the CPP.

The newly commenced REBOARREST trial is a randomised controlled trial (RCT) that aims to investigate the efficacy of REBOA as an adjunct treatment to advanced cardiovascular life support (ACLS) [12]. The current study was initiated to substantiate the physiological rationale for REBOA in non-traumatic cardiac arrest patients prior to this RCT. The aim of this study was to investigate the peripheral arterial pressure response to aortic occlusion during CPR in patients suffering from non-traumatic out of hospital cardiac arrest (OHCA).

## Methods

This was a prospective observational study, performed by the physician-staffed helicopter emergency medical service (HEMS) in Trondheim, Norway, with a catchment population of approximately 700,000. The personnel at the HEMS base has previously been educated in the use of the REBOA technique during ACLS [13]. This current study was an extension of a previously performed pilot feasibility study with the same ClinicalTrials.gov-reference, which reported on the use of REBOA in 10 patients [3]. Ethical approval was granted to include an additional 10 patients, and patient inclusion began in December 2019. Due to the Covid-19 pandemic, inclusion of patients was halted in most of 2020 until March 2021, and the study was prematurely stopped in April 2021 before the intended 10 patients had been included.

All patients that met inclusion/exclusion criteria were resuscitated on scene according to the current ACLS guideline published by the Norwegian Resuscitation Council [14].

Patients between 18 and 75 years of age, with non-traumatic cardiac arrest and by-stander CPR commenced within 10 min were included. Exclusion criteria were suspected or known pregnancy, known terminal illness, accidental hypothermia, drowning, strangulation and suspected intracerebral haemorrhage.

Patients were endotracheally intubated, manually ventilated and received mechanical chest compressions (LUCAS CPR, Physio Control-Inc, Lund, Sweden). Invasive arterial BP were measured via the left radial or brachial artery and registered at one-minute intervals. If the physician failed to achieve peripheral arterial access in the left radial or brachial artery, the patient was excluded and did not receive REBOA as an adjunct treatment, and standard ACLS was provided as per routine. The REBOA procedure was performed in sterile conditions under ultrasound guidance (iViz, FUJIFILM SonoSite, WA, USA) via the femoral artery. A catheter (7 Fr, 20 mm, Reboa Balloon Kit, Reboa Medical AS, Norway) was inserted to a length of 50 cm for an aortic zone 1 occlusion. The balloon was inflated with the amount of 0,9% saline specified by the producer or to resistance was felt, to ensure full aortic occlusion. The detailed procedure has previously been reported [3] and is available at www.reboarrest.com.

### Data collection

Demographic and cardiac arrest variables were obtained through a semi-structured interview with the performing physician and by the Utstein template for CPR related variables [15]. Time of HEMS dispatch, arrival, procedure duration and ROSC were obtained from the emergency medical communications central database and a specifically designed checklist chart.

End-tidal CO_2_ (EtCO_2_) values were obtained (Corpuls3, GS, Germany) and measured before balloon inflation, directly after inflation, 30, 60 and 90 s after inflation, and after ROSC of any duration. This was performed similar as to the pilot study [3]. Invasive arterial BP measurements were registered every minute and obtained from the Corpuls3. Due to this one-minute sampling rate, the BP 2 min before and after aortic occlusion was used in assessment of the primary outcome to ensure an appropriate interval before and after aortic occlusion.

### Statistical analysis

Data was analysed with IBM SPSS Statistics 27 and R version 3.6.0. The R package ‘ggplot2’ was applied for visualization. Continuous variables are reported as mean with standard error or median with range, as appropriate. Categorical variables are described as count and/or proportion. Differences in arterial BP and EtCO_2_ before and after occlusion was analysed with Wilcoxon Signed Rank test. A *p* value of < 0.05 was regarded as statistically significant.

## Results

**Fig. 1:**
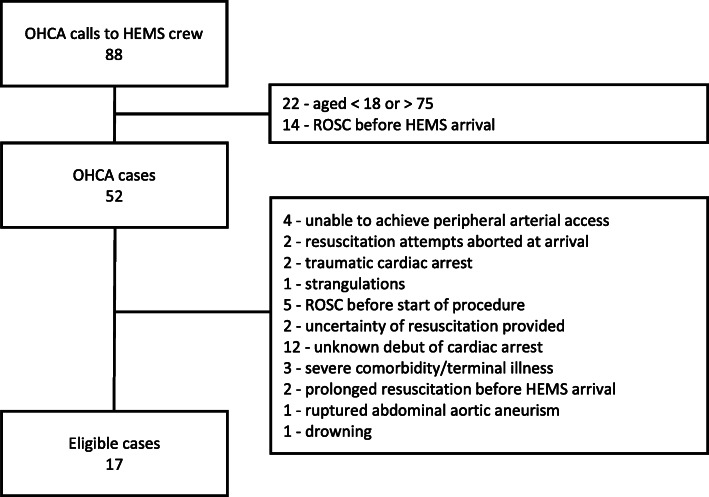
Flowchart of patients eligible for pre-hospital aortic occlusion with simultaneous peripheral invasive arterial blood pressure measurements during cardiac arrest. OHCA indicates out of hospital cardiac arrest; HEMS, helicopter emergency medical service; ROSC, return of spontaneous circulation

During the study period, HEMS was dispatched 88 times due to OHCA. Of these, 22 were older than 75 years and 14 had obtained ROSC before the arrival of the HEMS crew. Thirty-five patients were not eligible for REBOA due to other exclusion criteria (Fig. 1). In total, seven patients received REBOA as an adjunct treatment to ACLS. Two of these were subsequent excluded from the study due to extra-arterial placement of the peripheral arterial line. All procedures were performed indoors. The arterial pressure was measured in the left radial artery in four patients and in the left brachial artery in one patient. The REBOA procedure was successful in all five patients at first cannulation attempt. In all patients the cannulation was performed during a 10–20 s pause in chest compressions. No procedure complications such as excess bleeding at puncture site, equipment malfunction or resistance to introduction of the equipment occurred. Baseline details and relevant procedure data for the five patients are described in Table 1.

**Table 1:** Baseline characteristics and procedure data for the five patients. Time from dispatch to ROSC are only indicated for the two patients with ROSC. HEMS indicates helicopter emergency medical service; ROSC, return of spontaneous circulation

Baseline characteristics and procedure data	
Male, n (%)	5 (100)
Age, median (range)	63 (45–71)
Time of cardiac arrest	
Daytime (08–23), n (%)	3 (60)
First monitored rhythm by HEMS	
Asystole, n (%)	2 (40)
Pulseless electrical activity, n (%)	2 (40)
Ventricular fibrillation/tachycardia, n (%)	1 (20)
Dispatch to arrival on scene, median (range), min	29 (10–38)
Dispatch to occlusion, median (range), min	50 (39–72)
Dispatch to ROSC, mean (range), min	53.5 (50–57)

The median time from dispatch to aortic occlusion was 50 min (range 39–72). Two of the five patients achieved ROSC and one patient was admitted to hospital. No patients survived to day 30.

The mean EtCO2 value before start of aortic occlusion was 3.00 kPa and increased by a mean of 1.08 kPa (*p* = 0.104) 30 s after occlusion. From before start of aortic occlusion to 60 s after occlusion, the EtCO2 increased by a mean of 1.16 kPa (*p* = 0.043).

**Fig. 2:**
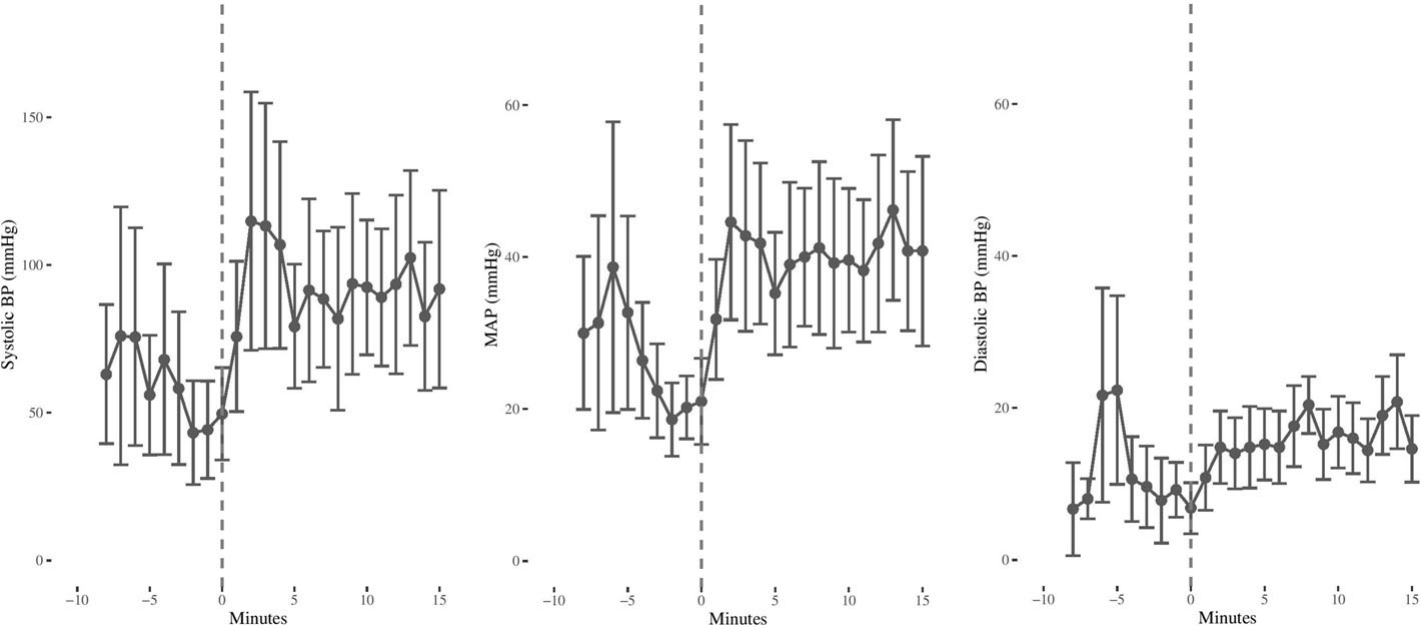
Peripheral blood pressure changes after aortic occlusion. Peripheral artery pressure changes after aortic occlusion, mean values +/− standard error. Occlusion is at 0 min. BP indicates blood pressure; MAP, mean arterial pressure

The peripheral artery pressures changes after aortic occlusion are demonstrated in Fig. 2 and Table 2. Arterial pressure 2 min before occlusion and 2 min after occlusion were significantly different in the compression phase (“systolic”) and as mean pressure and not significant in the relaxation phase (“diastolic”).

**Table 2:** Peripheral artery pressures before and after aortic occlusion

	Before aortic occlusion	After aortic occlusion	***p***-value
Compression phase pressure, mmHg (range)	43.2 (12–112)	114.8 (23–241)	0.043
Mean pressure, mmHg (range)	18.6 (4–27)	44.6 (15–87)	0.043
Relaxation phase pressure, mmHg (range)	7.8 (−7–22)	14.8 (0–29)	0.223

One patient showed signs of CPR-induced consciousness after aortic occlusion, to the extent that sedation was needed. The tertiary hospital was consulted for possible extracorporeal membrane oxygenation treatment, but it was declined due to long duration of cardiac arrest. The resuscitation efforts were then abandoned.

## Discussion

To our knowledge, this is the first study to report changes in peripheral arterial pressure due to aortic occlusion in humans suffering from OHCA. Our findings demonstrate that aortic occlusion during ACLS is associated with increase in peripheral arterial pressure. The REBOA catheter in use is not approved to perform aortic pressure recordings, hence we were not able to measure the central aortic blood pressure. However, we find it likely that an increase in the radial or brachial arterial pressures during CPR also indicate an increase in central aortic blood pressure.

Blood pressure can differ significantly between the central and peripheral arteries [16], but few studies describe simultaneous radial arterial and central aortic pressures during CPR. One human study demonstrated that the radial arterial pressure correlated with the aortic pressure during CPR [10] and another study found both a compression phase and relaxation phase gradient between the radial artery and the right atria [9]. In two case reports the radial arterial compression phase, relaxation phase and mean pressures as well as the CPP increased after aortic occlusion [4]. These studies indicate first that changes in radial arterial pressures may indicate changes in central aortic pressures, and second that aortic occlusion may increase aortic pressure and subsequently the CPP.

Additionally, few studies, and with small sample sizes, report intra-arterial BP differences between the brachial and radial artery [17]. It is reported that most patients have systolic radial arterial BP > 5 mmHg higher than brachial and as much as 14% of the patients have radial arterial systolic BP > 15 mmHg higher than brachial, the so-called “Popeye phenomenon” [17]. It is also shown that brachial cuff BP measurements systematically underestimate the true intra-arterial brachial pressure by 5.7 mmHg [18], which results in a potential difference from brachial cuff-measured systolic pressure and invasive radial pressure above 20 mmHg. It is therefore important to consider how and where arterial pressure is measured in clinical practice. In this study, pressure was measured in the radial artery in four patients and in the brachial artery in one patient, and the small sample size is hence insufficient to allow any group comparison of these pressures.

Finally, of the 17 potentially eligible patients, only seven patients received REBOA and it is difficult in retrospect to determine the reason for this lack of compliance to protocol. However, it is likely due to some of the expected and challenging pre-hospital environmental factors considered to complicate the procedure, such as low temperature, light conditions, wind, rain, and unsafe location.

### Limitations

First, this data must be regarded as preliminary findings due to a limited number of patients. Second, it was a single-centre study, with few physicians and paramedics involved. Third, all the physicians were board-certified anaesthesiologist with considerable experience with the use of ultrasound and Seldinger technique, and the results may not be relevant to other settings. Fourth, the arterial pressures are measured with one-minute sampling rate. The BP may vary during this time interval, hence further studies may benefit from the use of higher sampling rate, or continuous sampling. Finally, this study primarily increase knowledge on the hemodynamic changes caused by REBOA during CPR and cannot conclude about the potential clinical benefit from a REBOA intervention.

## Conclusion

To our knowledge, this is the first study to investigate the peripheral invasive arterial blood pressure response to aortic occlusion during non-traumatic OHCA patients. REBOA as an adjunct treatment during resuscitation may significantly increase the peripheral artery pressures and it is likely that this indicates improved central aortic blood pressure. Our findings warrant studies with simultaneous peripheral and central blood pressure measurement during aortic occlusion.

## Data Availability

The datasets used and/or analyzed during the current study are available from the corresponding author on reasonable request.

## Abbreviations

ACLS: Advanced cardiovascular life support
BP: Blood pressure
CPP: Coronary perfusion pressure
CPR: Cardiopulmonary resuscitation
EtCO_2_: End-tidal CO_2_
HEMS: Helicopter emergency medical service
OHCA: Out of hospital cardiac arrest
RCT: Randomised controlled trial
REBOA: Resuscitative endovascular balloon occlusion of the aorta
ROSC: Return of spontaneous circulation
